# Papillary thyroid carcinoma with massive metastasis in the uterine corpus: a case report

**DOI:** 10.1186/1471-2407-13-551

**Published:** 2013-11-19

**Authors:** Jian-hong Wang, Jing Yu, Chun-ping Ning, Yong-mei Sun, Shi-bao Fang

**Affiliations:** 1 Ultrasound Department, Hospital of Qingdao University, Medical College, Qingdao, China, No. 16 Jiangsu Road, Southern Distinct of Qingdao, Shandong Province, China

**Keywords:** Papillary thyroid carcinoma, Metastasis, Ultrasound, Uterine

## Abstract

**Background:**

Distant metastases stemming from a papillary thyroid carcinoma (PTC) are quite rare. Here we report an exceptional case of PTC presenting with cervical lymphatic and uterine metastases. This is the first case report of a PTC with uterine involvement.

**Case presentation:**

A 60-year-old Chinese woman came to our hospital complaining of discomfort in the throat that she had been experiencing for about half a month. PTC and cervical lymphatic metastasis were diagnosed after ultrasound examinations. A massive heterogeneous mass was found beside the uterus during the pre-operative checkup and a diagnosis of ovarian carcinoma was suspected after a thorough case discussion. However, it proved to be a metastasis from the PTC as determined by pathological and immunohistochemical examinations after the operation. The patient declined further treatments. She was followed for 22 months with no sign of recurrence detected.

**Conclusions:**

In this report, an unusual case of PTC was presented. The patient had not only regional lymphatic metastasis, but also had a massive metastasis in the uterine corpus, which was initially misdiagnosed as ovarian carcinoma. This case is of interest because of its rarity and exceptionally good prognosis. The reason for the misdiagnosis was attributed to overlooking the possibility of a distant metastasis coming from a PTC. This case raises the issue that completing an iodine-131 scan before operating on patients with PTC may be warranted.

## Background

Papillary thyroid carcinoma (PTC) is the most commonly seen well-differentiated malignancy that generally has a good prognosis. Regional metastases usually involve cervical and upper mediastinal lymph nodes. Distant metastases, although quite rare, have a profound impact on survival. The reported 5-year survival rate of PTC patients operated on for a distant metastasis was approximately 55.0% [1].

A pulmonary metastasis was recognized as the dominant site for a distant metastasis of PTC, followed by bone [2]. Brain, skin, liver and kidney [3] were seldom involved and occurrence of a uterine metastasis was exceptional. We report herein a patient with PTC with cervical lymphatic metastasis accompanied by uterine metastasis. It is of interest for its rarity and exceptionally good prognosis.

## Case presentation

### Case report

**Figure 1:**
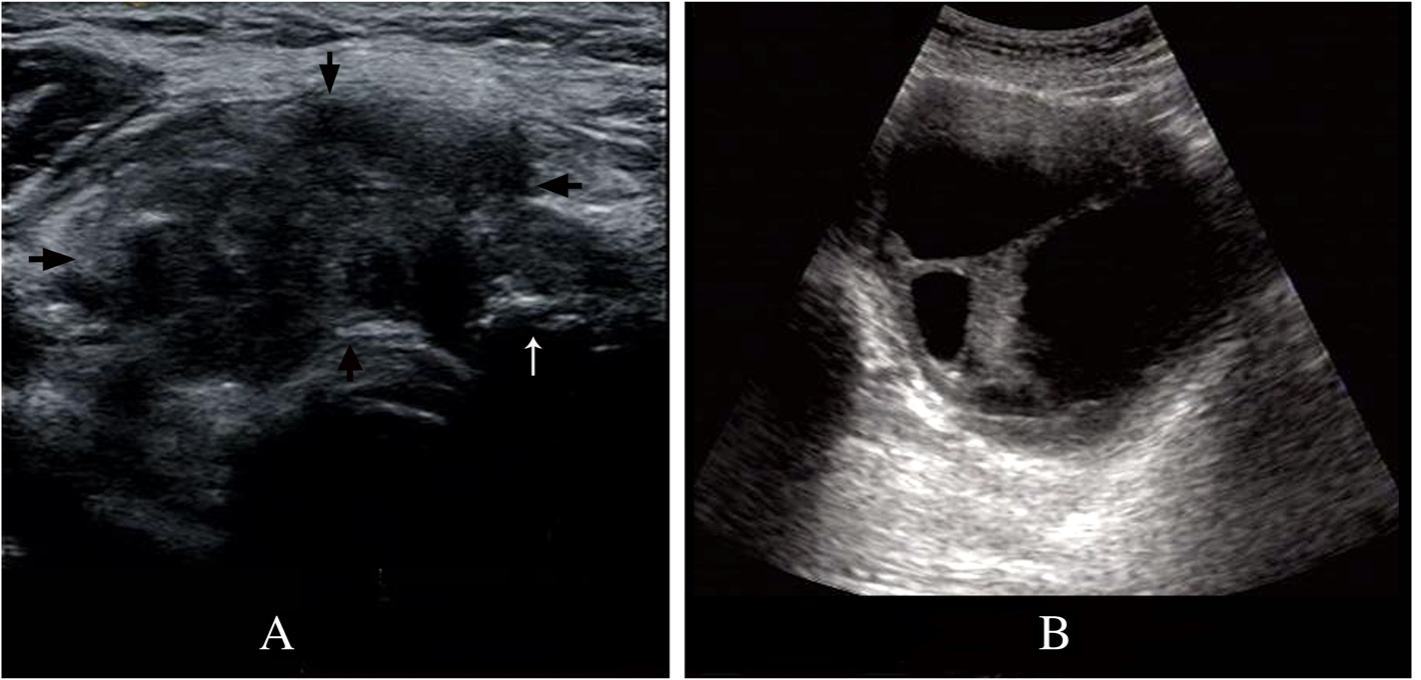
**Ultrasound images of the thyroid nodule and the uterine mass. A**. A heterogeneous hypoechoic mass was detected in the thyroid. The blurred margins are marked by four arrowheads (→). Coarse calcifications with heavy echoic shadow are highlighted by a white arrow (↑). **B**. A large mass, containing a sizeable portion of fluid and thick separations, was revealed by ultrasound examination during the pre-operative checkup.

A 60-year-old woman presented to our hospital on September 6, 2011, complaining of discomfort in the throat that she had been experiencing for about half a month. Past medical history was not a factor and there was no family history of thyroid cancer either. Physical examination revealed a palpable nodule in the right lobe of the swollen (grade I) thyroid. An ultrasound examination was performed immediately and an irregular hypoechoic nodule (measuring 31 × 15 mm) was detected arising from the lower pole and extending to the isthmus of the thyroid. The nodule was solid with blurry margins (Figure 1A) and it was difficult to distinguish from the trachea. Several coarse calcifications with heavy shadows were noticed inside. The thyroid capsule was also involved. Two swollen lymph nodes were found beside the right internal jugular vein (level V) measuring 17 × 13 mm and 14 × 10 mm, respectively. Computed tomography (CT) scans of the lungs were done but no positive findings were noted.

Because invasive thyroid carcinoma with cervical lymphatic metastases was assumed, surgical treatment was scheduled. Before the operation, the patient received a general checkup, including blood tests, echocardiographic assessment, abdominal and gynecologic ultrasound examinations, and CT scans of the head and abdomen. A heterogeneous mass was found in the right lower quadrant behind the uterus. The mass, measuring 115 × 92 × 94 mm, contained a large quantity of echoic fluid. Thick separations and two irregular solid components (measuring about 33 × 37 mm and 33 × 21 mm, respectively) were noticed inside. The ultrasound image is shown in Figure 1B. Abundant blood flow signals were detected in the solid components using color Doppler. The outside border of the mass was quite clear and smooth, but inside the mass closely adhered to and was difficult to distinguish from the right wall of the uterus. Neither ovary was involved. The serum cancer antigen 125 (CA125) level was slightly elevated at 70.58 U/ml (normal range, 0-35 U/ml). Carcinogenic embryonic antigen (CEA) (1.97 ng/ml) and CA19-9 (8.82 U/ml) levels were normal. Given the complex texture of the mass, an ovarian tumor, probably a cystadenocarcinoma, was suspected. After a thorough discussion with the relatives of the patient, a second operation following treatment of the thyroid nodule was scheduled.

**Figure 2:**
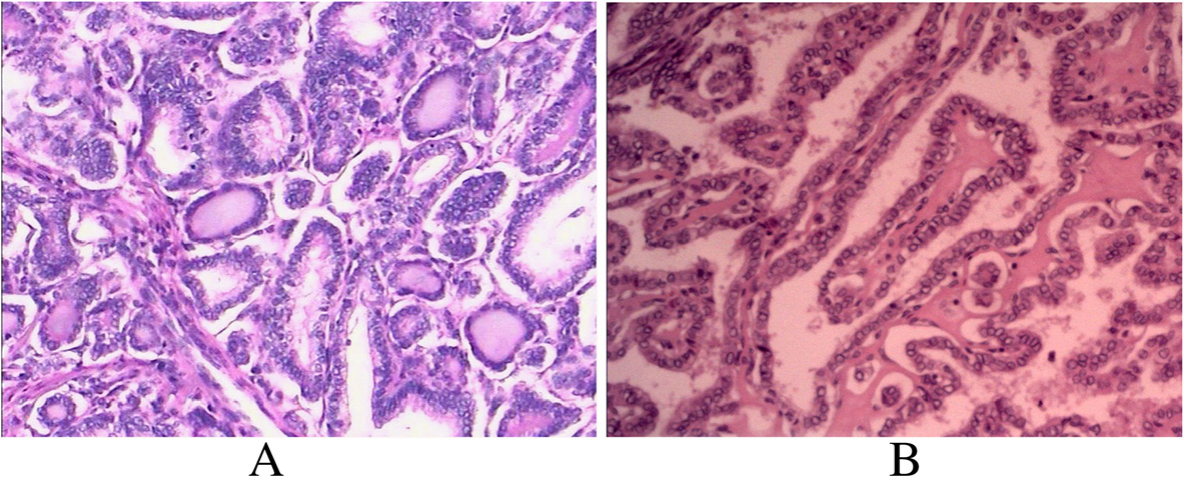
**Pathological images of the thyroid and uterine masses.** A pathological image of the heterogeneous mass in the thyroid and uterus is shown in **A** and **B**, respectively. Both of them were stained with hematoxylin and eosin and the image was magnified 40 times. The classical papillary growth pattern can be seen in both masses.

A total thyroidectomy and neck exploration were performed on September 17, 2011, under general anesthesia. Histological examination confirmed the diagnosis of a well-differentiated PTC (Figure 2A). Immunohistochemical examination demonstrated that the cells stained positively for thyroid transcription factor (TTF), cyclin D1, P27, epidermal growth factor receptor (EGFR) and Ki-67 (ratio 10%), and were negative for cytokeratin 19. Regional lymph node (3/4) metastases were confirmed. The patient had an uneventful postoperative course and was discharged on the 7^th^ postoperative day.

Fifteen days later she returned to our department of gynecology for further treatment of the abdominal mass. An abdominal exploration was performed and a large pink mass was detected arising from the posterior wall of the uterus. The mass was soft and covered by the serosa of the uterus. Both ovaries were easily identified because they were separated from the uterus and had a normal appearance. No ascites was found in the pouch of Douglas. A total abdominal hysterectomy along with bilateral salpingo-oophorectomy, omentectomy and a periaortic lymph node biopsy were performed while taking into consideration the age of the patient. On gross sections, several cysts were revealed that were filled with brown fluid inside the large heterogeneous mass. The two irregular solid components shown on the sonograms were also seen on the inside wall of the mass, and both of them had a gray and granular surface. No evidence of malignant changes was detected in tissue slices taken from the ovaries.

Postoperative pathological results were available five days later, and the diagnosis of a uterine metastasis stemming from the PTC was suspected (Figure 2B). To rule out a primary uterine carcinoma, an immunohistochemical assay on an appropriate panel of proteins was performed. The heterogeneous mass showed strong expression of TG, CK7, CAM5.2 and TTF. Results for CA125, EMA and CD10 were negative. Therefore, the diagnosis of metastatic PTC in the uterine corpus was established. No positive findings resulted from the harvested lymph nodes. The clinical course following surgery was indolent; the patient was discharged on October 14, 2011. Just prior to being discharged, she accepted to undergo a general radioactive iodine scan, and no abnormal hotspots were found. For unclear reasons, the patient declined further treatments and follow-up was suggested. Since then, she has returned three times for general checkups and once for a PET-CT examination, and thus far no signs of recurrence have been detected.

## Discussion

The uterus, mostly composed of smooth muscle tissue, has rarely been identified as a metastatic site of extragenital primary cancers [4]. When this occurs, the ovaries and endometrium are most often affected (75-80% of the cases), with breast and gastrointestinal carcinomas [5,6] being the most common primary extragenital cancers.

PTC, which corresponds to approximately 65-80% of thyroid tumors, is usually confined to the gland or tends to metastasize locally to mainly regional lymph nodes [7]. Distant metastasis of PTC is rare, but of the utmost importance for the patient, as the impact on prognosis is substantial. According to the literature, the most frequent sites for PTC distant metastases are lungs (72-76% of cases), mediastinum (24%) and bones (19-23%), while there are only sporadic reports of distant spread to other locations. Dr. Kumar described a case of medullary thyroid cancer with a uterine metastasis, but no detailed clinical or radiological information was provided [8]. Here, we have described a rare and perhaps the first case of a PTC metastasis restricted to the uterine corpus without involving the ovaries.

The patient had no obvious gynecologic symptoms when she came to our hospital. The metastatic mass was detected during a pre-operative checkup. On sonograms, it was heterogeneous with thick separations and solid components, mimicking an ovarian mucinous cystadenocarcinoma. Because of the patient’s age and the shielding afforded by the large mass, neither ovary was detected before the operation. Consequently, the mass was at first misdiagnosed as an ovarian cancer. Considering the volume of the mass, all participants in the treatment group agreed that the uterine metastasis most probably occurred much earlier than the detection of the thyroid nodule. The potential mechanism that could account for this was discussed. As there were no abnormal hot spots found on the radioactive iodine scan after the second operation, and no positive findings in the harvested abdominal lymph nodes, we believe that the metastasis was hematogenously disseminated. Though very rare, the possibility of malignant transformation of the struma uteri [9] was also proposed and then retracted because no normal thyroid tissue was found in the tumor or in the corpus of the uterus.

In terms of treatment for patients with PTC who have distant metastases, no standard protocol has been proposed. It was reported that radioiodine ablation cures only 7% of patients with bone metastases [10]. Researchers postulated that metastases from a differentiated thyroid cancer may not concentrate radioactive iodine because the cells were more immature than the original thyroid neoplasm. Here, the patient accepted only surgical resection of the tumor and hence, no further treatments were performed. Despite this, the patient has already lived an additional 22 months post-surgery with no sign of recurrence. We attribute the good prognosis to the high degree of differentiation of the primary carcinoma.

## Conclusions

To our knowledge, this is the first case report of a PTC with synchronous secondary uterine involvement. This case raises a discussion on dealing with differential diagnostic problems in the setting of clinical and pathological investigations of patients with PTC. The possibility of a uterine metastasis, as described here, may warrant completing an iodine-131 scan before operating on patients with PTC.

### Consent

Written informed consent was obtained from the patient for publication of this Case report and any accompanying images. A copy of the written consent is available for review by the Editor of this journal.

## Competing interests

This study was partially funded by the Municipal Science Support Plan (No. 13-1-3-36-nsh). The case was one of the special cases in the program entitled ‘Study of the Prevention of Transference of Papillary Thyroid Carcinoma in Qingdao District’. The authors have no competing interest, including relevant financial interests, activities, and affiliations.

## Authors’ contributions

SF, guarantor of integrity of the entire study. JW, study concepts and design. JY, CN literature research. JW clinical studies. CN, JY, manuscript preparation. YS, manuscript editing. All authors read and approved the final manuscript.

## Pre-publication history

The pre-publication history for this paper can be accessed here:

http://www.biomedcentral.com/1471-2407/13/551/prepub
